# A Conservatively Managed Giant Ascending Aortic Aneurysm Presenting With New Atrial Fibrillation and Heart Failure

**DOI:** 10.7759/cureus.116519

**Published:** 2026-09-18

**Authors:** Alyaa Hussein, Mohamed Ali, Diane James, Hamza Hamza, Martin Behounek

**Affiliations:** 1 Cardiology, University Hospitals of Leicester NHS Trust, Leicester, GBR; 2 Respiratory Medicine, Glenfield Hospital, University Hospitals of Leicester NHS Trust, Leicester, GBR; 3 Respiratory Medicine, University Hospitals of Leicester NHS Trust, Leicester, GBR

**Keywords:** aortic regurgitation, ascending aortic aneurysm, atrial fibrillation, clinical frailty scale, conservative management, decompensated heart failure, direct oral anticoagulant, giant aortic aneurysm, shared decision-making

## Abstract

Ascending aortic aneurysms bigger than 9 cm are not common these days. Most are found and fixed while they are still much smaller. We report an 88-year-old woman who came into hospital with palpitations, worsening breathlessness on exertion, and swollen ankles. She had a history of high blood pressure, stage 3 kidney disease, and high cholesterol. On examination, her pulse was irregular, she had crackles at the bottom of both lungs, and swelling up to her knees. Her ECG showed atrial fibrillation with a fast heart rate, and her B-type natriuretic peptide (BNP) blood test was very high at 4544 ng/L. She was treated for heart failure caused by the new atrial fibrillation and started on medicine to slow her heart rate and thin her blood. An echo scan, done just to check how well her heart was pumping, picked up something unexpected: the main part of her aorta near the heart was very dilated, there was leaking through the aortic valve, and there was fluid around the heart. A CT scan confirmed a 9.3 cm aneurysm of the ascending aorta, starting at the root, with no tear or bleeding. The heart surgeons felt she was too old and frail, with too many other health problems, to have surgery to fix it. She was treated for decompensated heart failure occurring in the setting of new-onset atrial fibrillation with a rapid ventricular response and was started on rate-control therapy and anticoagulation. We talked to her about whether to keep her on blood thinners even though the aneurysm could not be repaired. She chose to stay on her blood thinner after the risks were explained to her. She went home after five days and was doing better when seen again two months later, and the aneurysm had not grown. This case shows how much a routine echo can find, and how hard the decision about blood thinners can be when there is an aneurysm that cannot be operated on.

## Introduction

Thoracic aortic aneurysms are frequently asymptomatic and may be discovered incidentally during imaging performed for an unrelated clinical problem [1]. As aneurysm size increases, so does the risk of dissection, rupture, and death. For patients with an ascending aortic aneurysm, elective surgical repair is generally considered when the aortic diameter reaches approximately 5.5 cm, with earlier intervention in selected patients who have additional high-risk features, such as a connective tissue disorder, a bicuspid aortic valve, rapid aneurysm growth, or other factors associated with an increased risk of aortic complications [1]. These recommendations must nevertheless be interpreted alongside the patient’s operative risk. In older adults with frailty, renal impairment, ventricular dysfunction, and multiple comorbidities, the decision to pursue repair requires an individualized assessment of the expected benefits and risks rather than reliance on aneurysm size alone.

Aneurysms measuring 9 cm or more are uncommon in contemporary practice because most are detected and treated at considerably smaller diameters. The term “giant” is not used uniformly in the literature, although it is commonly applied to aneurysms exceeding 9 or 10 cm [2-4]. The coexistence of a giant ascending aortic aneurysm, new-onset atrial fibrillation, aortic regurgitation, and decompensated heart failure presents a particularly complex clinical situation [1]. Enlargement of the aortic root may contribute to aortic regurgitation, increasing the volume load on the left ventricle and worsening heart failure [1]. Rate-control therapy must also be selected carefully in a patient with ventricular dysfunction and significant valvular regurgitation; the use of digoxin requires particular caution in older patients with chronic kidney disease, while beta-blocker therapy in significant aortic regurgitation remains a clinically nuanced decision [5-7]. Although anticoagulation is generally used in atrial fibrillation to reduce the risk of thromboembolic stroke, clinicians must consider the competing risks and potential consequences of aneurysm rupture [8].

We report this case because it brings together three clinically important features: an extreme ascending aortic aneurysm measuring 9.3 cm, incidental detection during evaluation of new-onset atrial fibrillation and decompensated heart failure, and nonoperative management in a frail older patient. The case also illustrates the competing risks involved in deciding whether to continue anticoagulation when definitive surgical repair is not an option. It therefore highlights the importance of multimodality imaging, individualized assessment of operative risk, and shared decision-making in the management of patients with complex cardiovascular disease.

## Case presentation

Presentation and Initial Assessment

An 88-year-old woman with known hypertensive and chronic kidney disease stage 3 came to the hospital with a few days of palpitations, worsening breathlessness, and swollen ankles. She had a history of high blood pressure, stage 3 kidney disease, and high cholesterol. She used to smoke but had stopped; she was not diabetic, she had no known drug allergies, and her memory and thinking were normal. Before this admission, she was fully independent and looked after herself.

On examination, her heart rhythm was irregular and fast. She had crackling sounds at the bottom of both lungs and swelling in both legs up to the knees.

Blood tests showed a very high B-type natriuretic peptide (BNP) level of 4544 ng/L, a hormone that goes up when the heart is under strain. Her white blood cell count, thyroid hormone, and magnesium were all normal. Her kidney function was in keeping with her known kidney disease. ECG showed new atrial fibrillation (Figure 1). Her chest X-ray (CXR) showed a bent, twisted aortic arch and what looked like a large mass in the middle of the chest (Figure 2).

**Figure 1:**
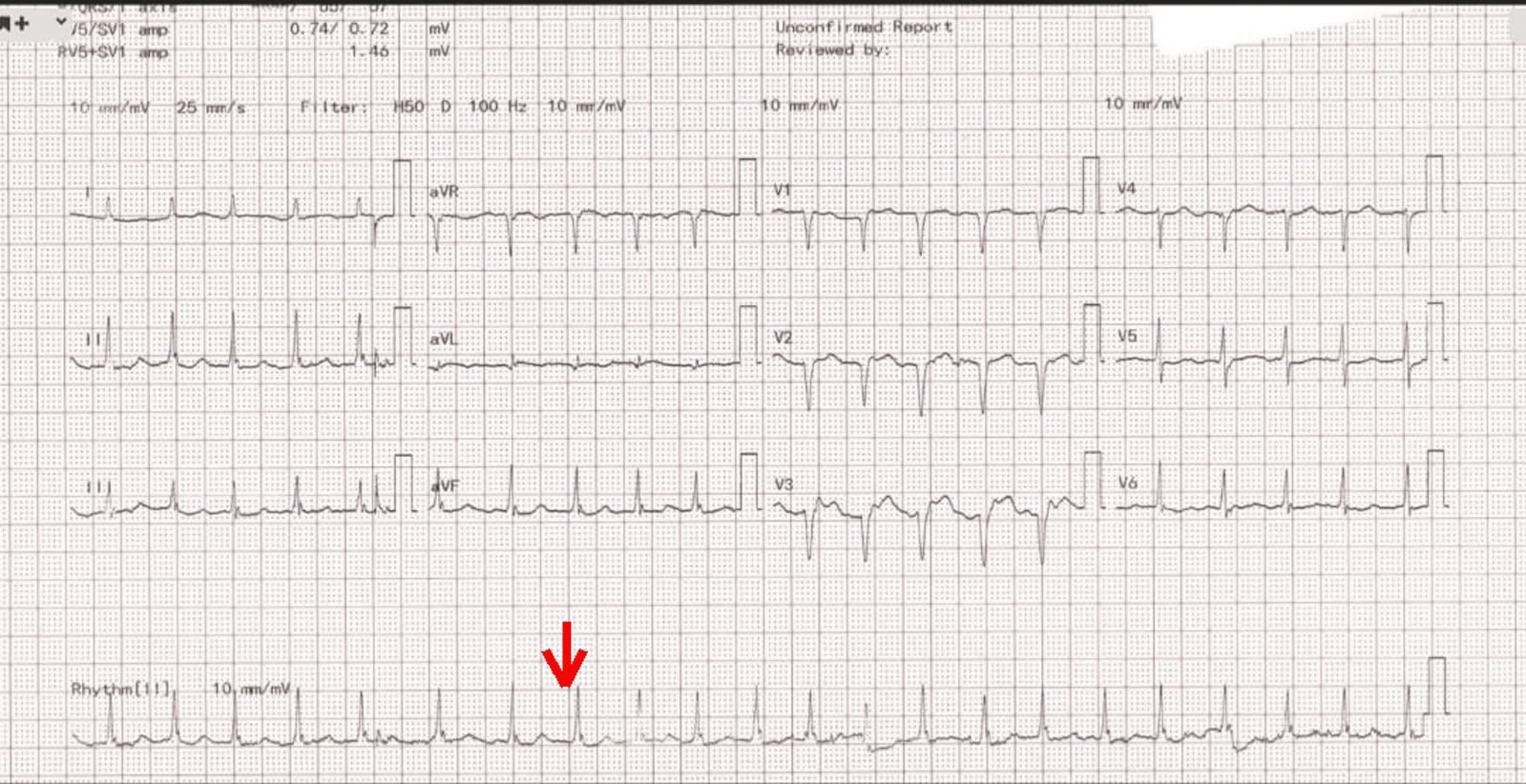
The ECG confirmed atrial fibrillation with a fast heart rate.

**Figure 2:**
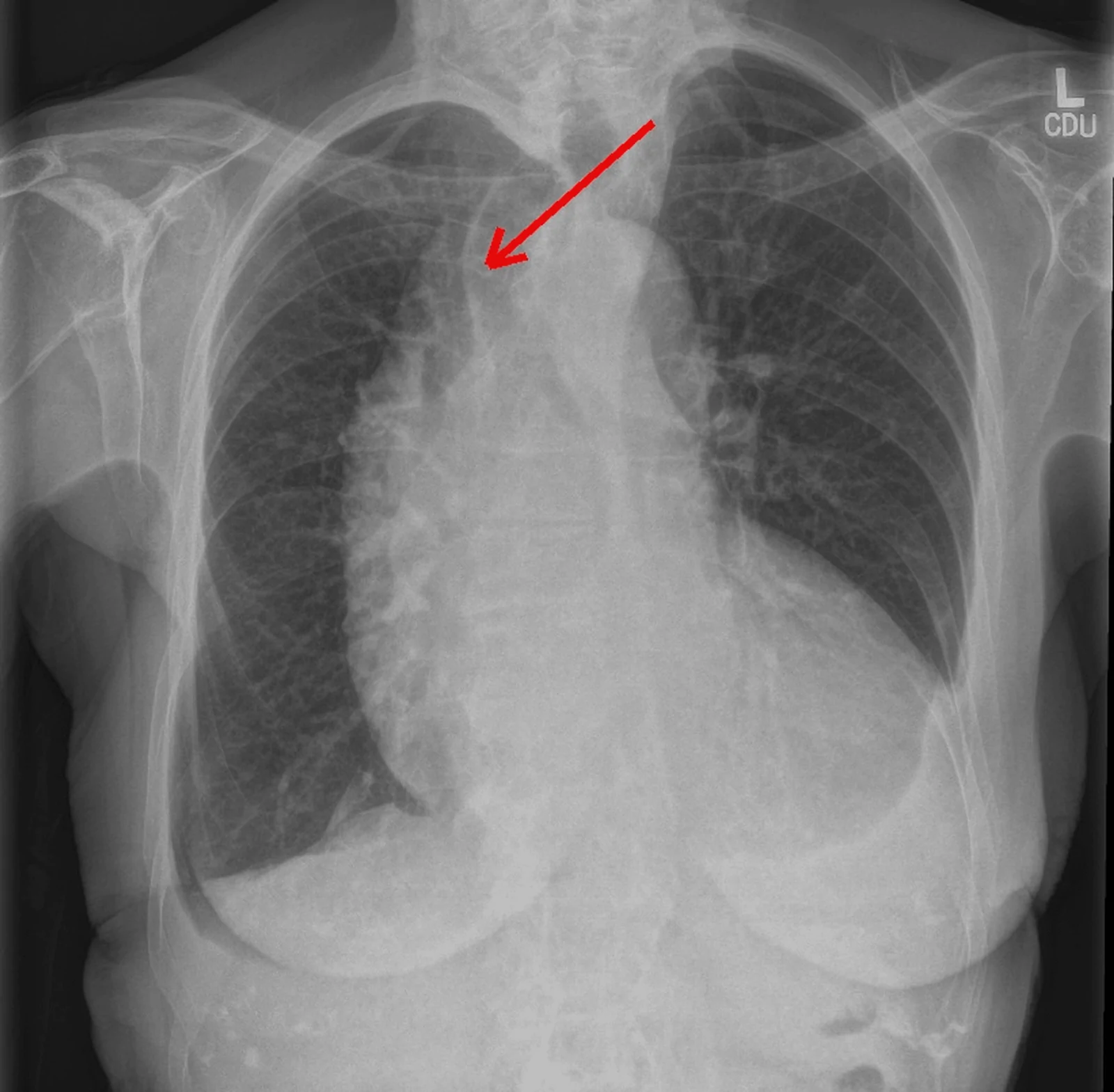
CXR showed a twisted aortic arch and what looked like a large mass in the middle of the chest.

Initial management

She was diagnosed with decompensated heart failure occurring in the setting of new-onset atrial fibrillation with a rapid ventricular response. The heart failure was considered multifactorial, with possible contributions from the rapid ventricular rate, significant aortic regurgitation, and underlying ventricular dysfunction. She was given digoxin to slow her heart rate, her bisoprolol dose was increased, and she was started on apixaban, a blood thinner, because of her risk of stroke. She was also given water tablets for the fluid overload.

Identification of the aneurysm

Transthoracic echocardiography was performed to assess ventricular function and evaluate the cardiac valves. Image quality was fair. The study showed marked dilatation of the proximal ascending aorta, measuring at least 7.5 cm in the available view. However, the aortic walls could not be traced reliably, and the echocardiographer appropriately noted that the measurement was limited and recommended computed tomography for definitive assessment (Figure 3). In the limited echocardiographic views, the aortic root appeared to be of normal caliber; however, visualization of the aortic root and the distal ascending aorta was incomplete, and the aortic walls could not be traced reliably. CT angiography subsequently demonstrated that the aneurysm arose at the level of the aortic root and extended through the ascending aorta. The CT findings were considered definitive for determining the anatomical origin and extent of the aneurysm.

**Figure 3:**
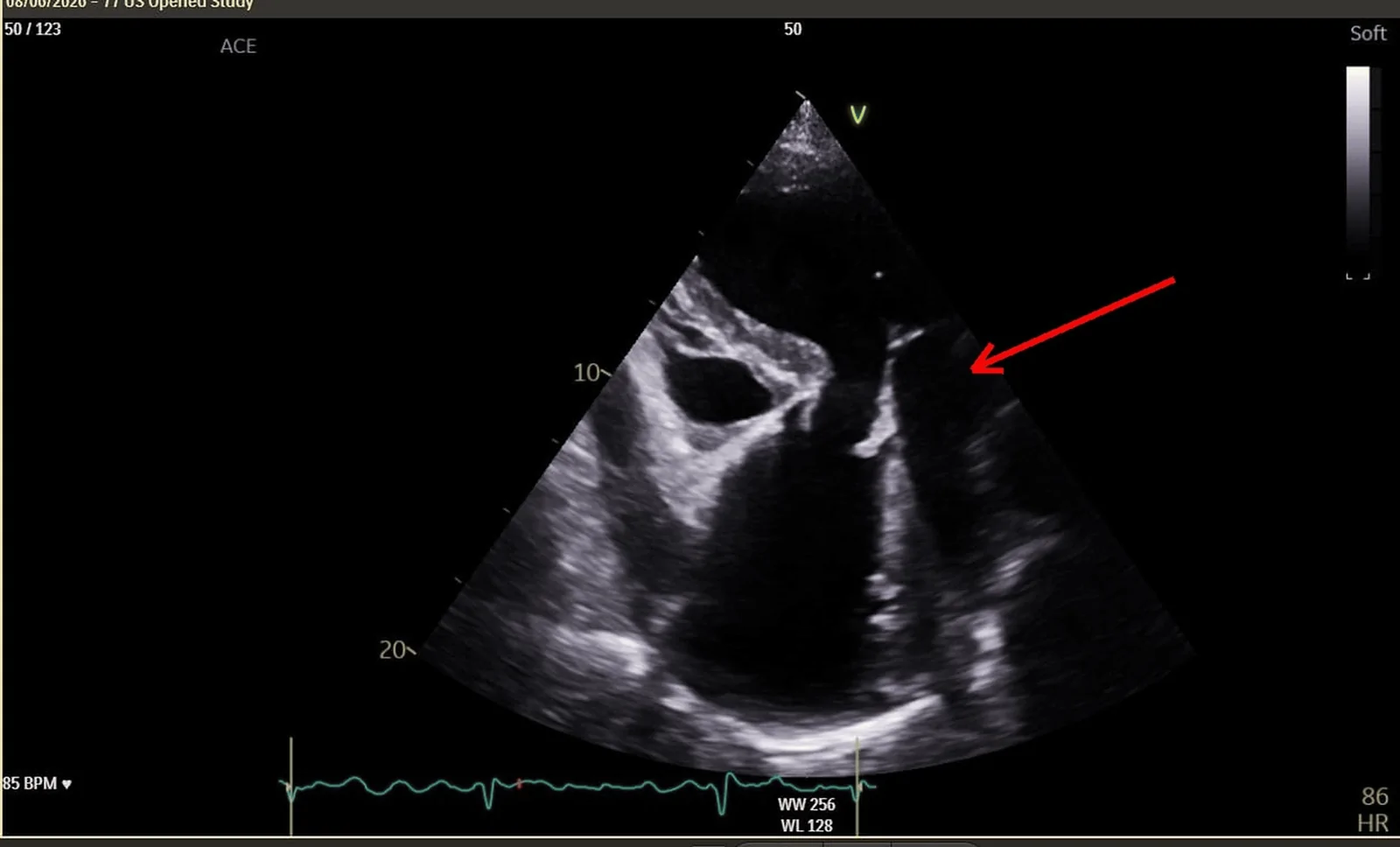
Parasternal long-axis echocardiogram showing the dilated ascending aorta.

A further attempt was made to measure the ascending aorta, but the aortic margins remained incompletely visualized. As a result, the echocardiographic measurement could not be considered definitive (Figure 4).

**Figure 4:**
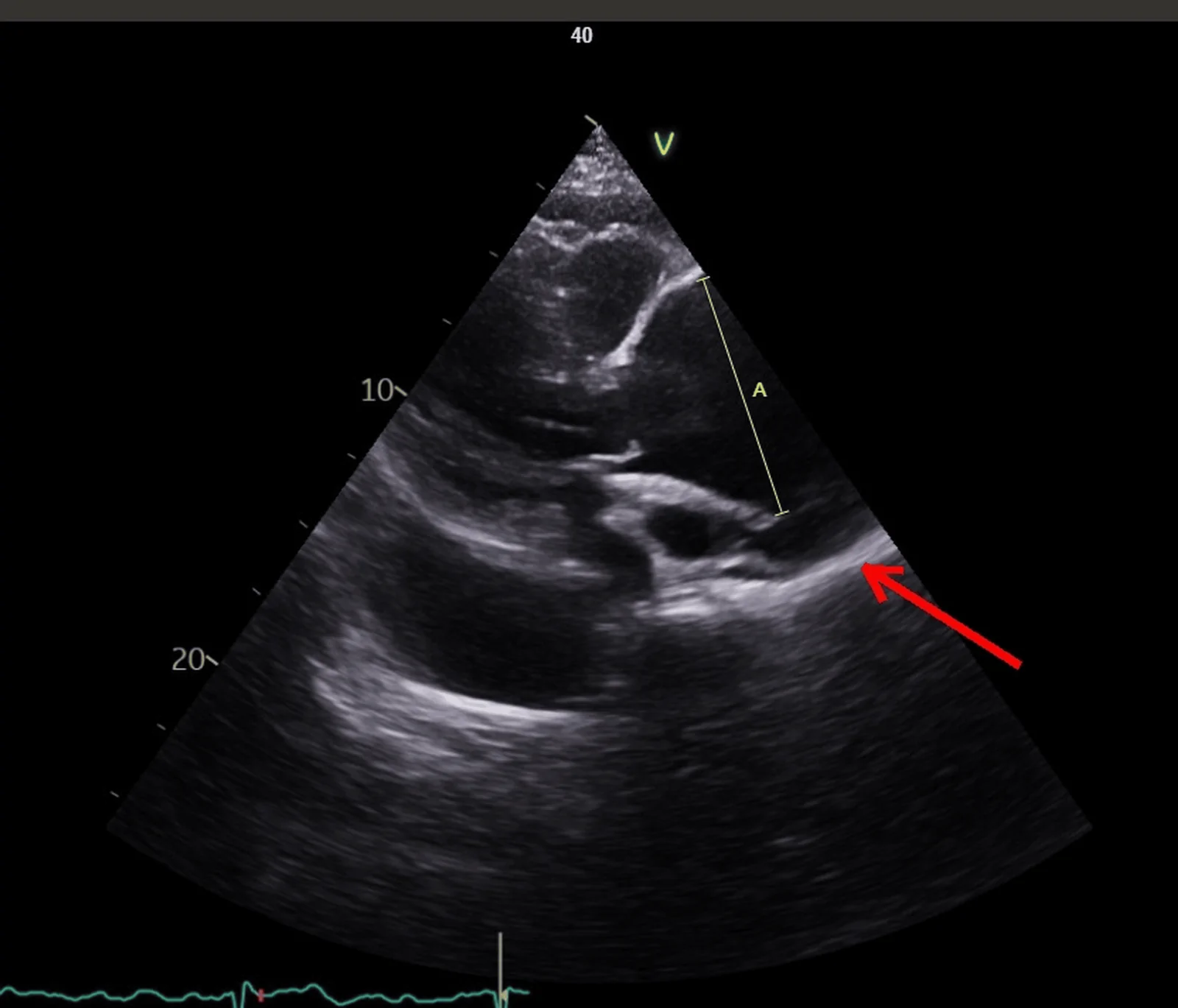
Echocardiographic attempt at measuring the ascending aortic diameter.

The aortic valve was trileaflet, mildly thickened, and opened well without stenosis. At least moderate aortic regurgitation was present and appeared closer to severe on some cardiac cycles. The regurgitant jet extended at least halfway into the left ventricular outflow tract. The vena contracta measured approximately 0.55 cm, and the jet width was approximately 45% of the left ventricular outflow tract width (Figures 5-6).

**Figure 5:**
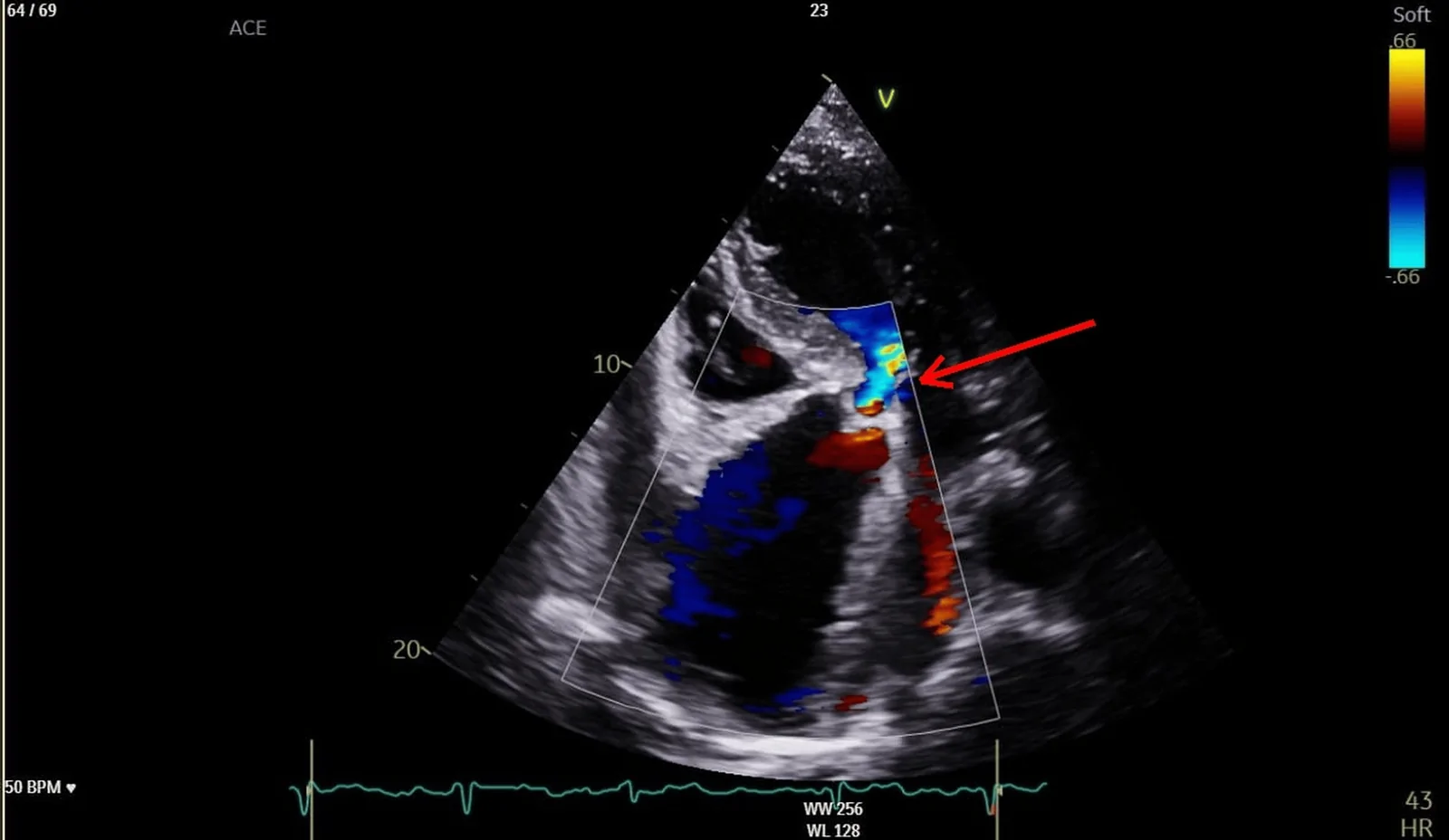
Colour Doppler echocardiogram showing the aortic regurgitant jet.

**Figure 6:**
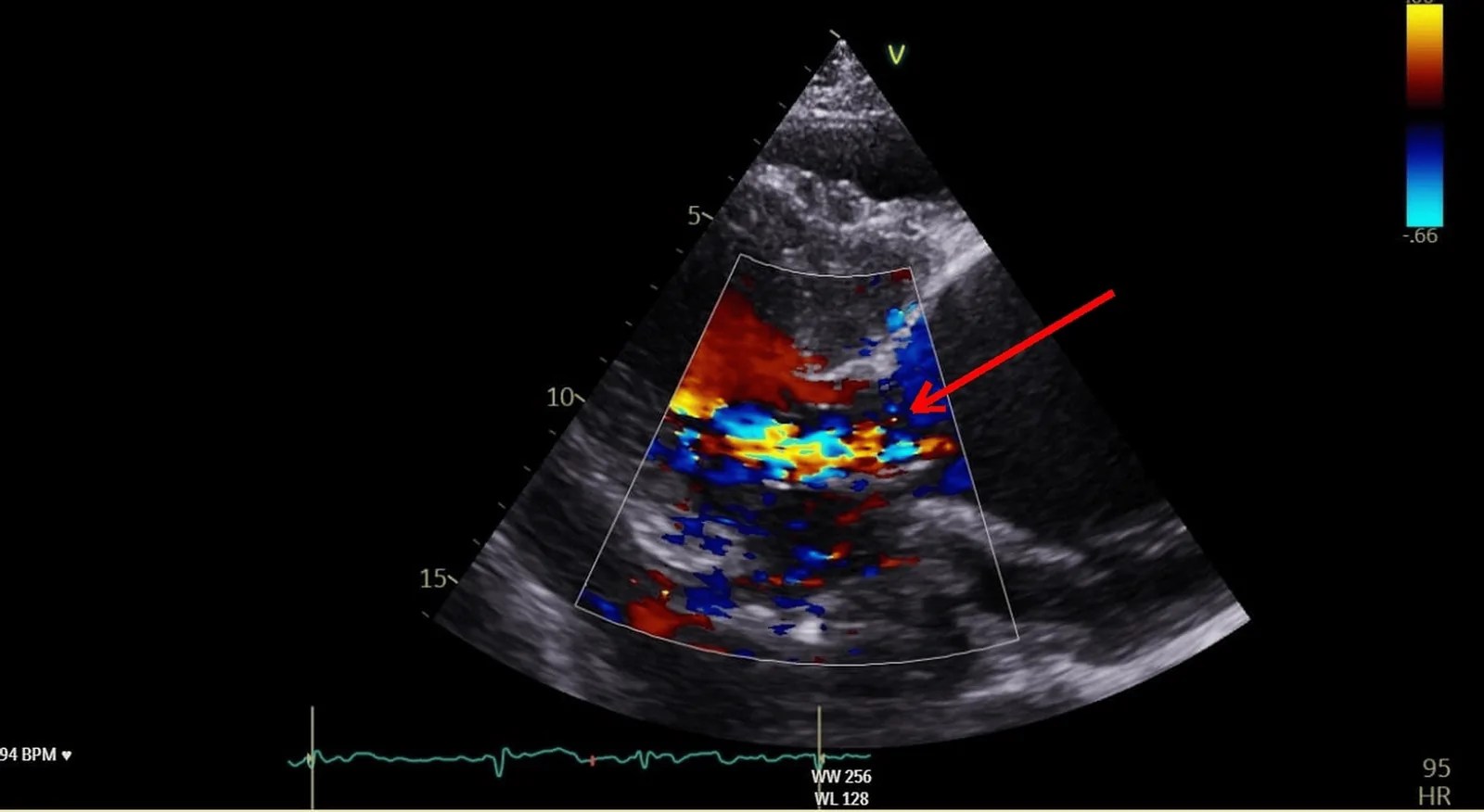
Magnified colour Doppler view of the aortic regurgitant jet

The left ventricle was normal in size, with mild concentric wall thickening, globally reduced wall motion, and a left ventricular ejection fraction of 40%. Left ventricular filling pressure could not be assessed reliably. The right ventricle was normal in size but mildly impaired in systolic function, with a tricuspid annular plane systolic excursion of 1.6 cm. Mild mitral, tricuspid, and pulmonary regurgitation was also present. The left atrium was enlarged, and the right atrium appeared enlarged in some views. Estimated pulmonary artery systolic pressure was 19.8 mmHg plus right atrial pressure. The inferior vena cava was normal in appearance.

A pericardial effusion was present, with an internal echogenic component measuring approximately 1.4 cm. There was no echocardiographic evidence of tamponade. A left-sided pleural effusion was also identified, with a possible small right-sided effusion.

Given the marked aortic dilatation and the limited echocardiographic visualization, contrast-enhanced CT angiography of the entire aorta was performed (Figures 7-10). This confirmed a giant ascending aortic aneurysm arising at the level of the aortic root and measuring 9.3 cm at its maximum diameter. A repeat review by a senior clinician measured the aneurysm at 9.2 cm. The aortic arch, descending thoracic aorta, and abdominal aorta were normal in caliber. There was no evidence of dissection, intramural hematoma, or rupture, and the major branch vessels were unremarkable. The CT scan also showed a moderate simple pericardial effusion measuring up to 2.5 cm, small bilateral pleural effusions, and dependent bibasilar atelectatic change. Sigmoid diverticulosis was noted incidentally. The abdominal viscera appeared normal, without hydronephrosis or suspicious osseous lesions. Two small splenic artery aneurysms were also seen incidentally; because of their size, no additional follow-up was recommended.

**Figure 7:**
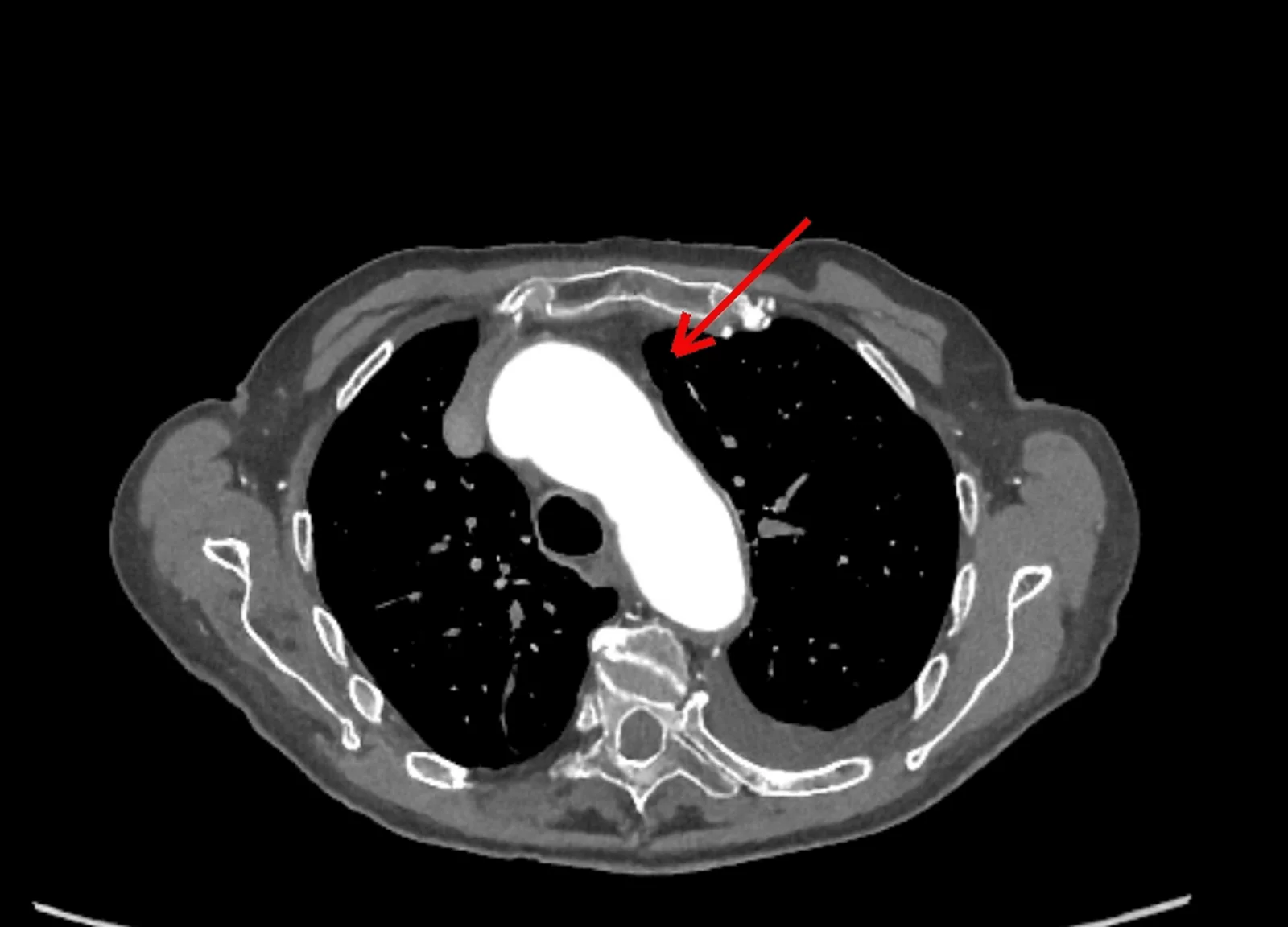
Axial CT angiogram at the level of the aortic root showing the giant ascending aortic aneurysm at its maximal diameter.

**Figure 8:**
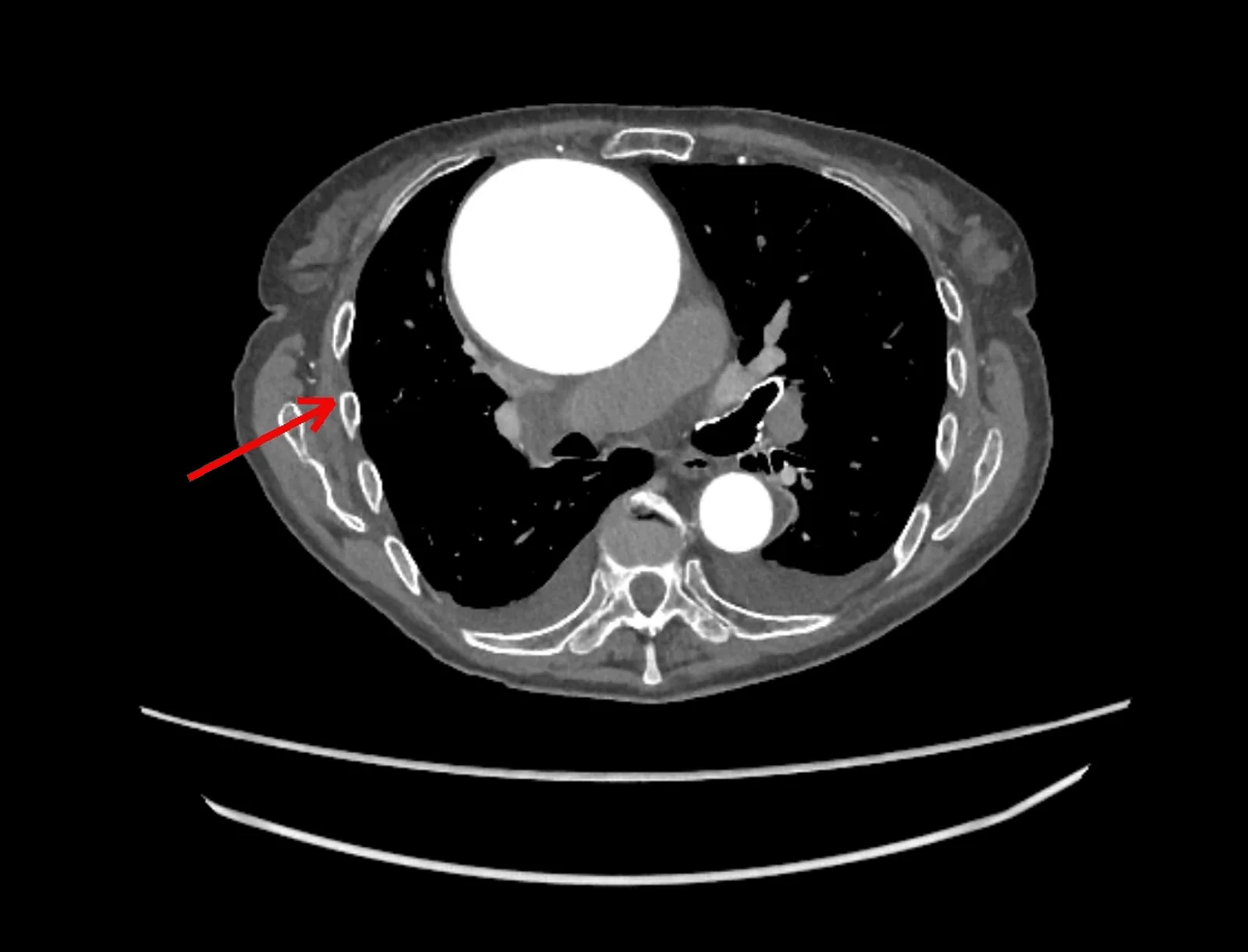
Axial CT angiogram showing the ascending aortic aneurysm with bilateral pleural effusions.

**Figure 9:**
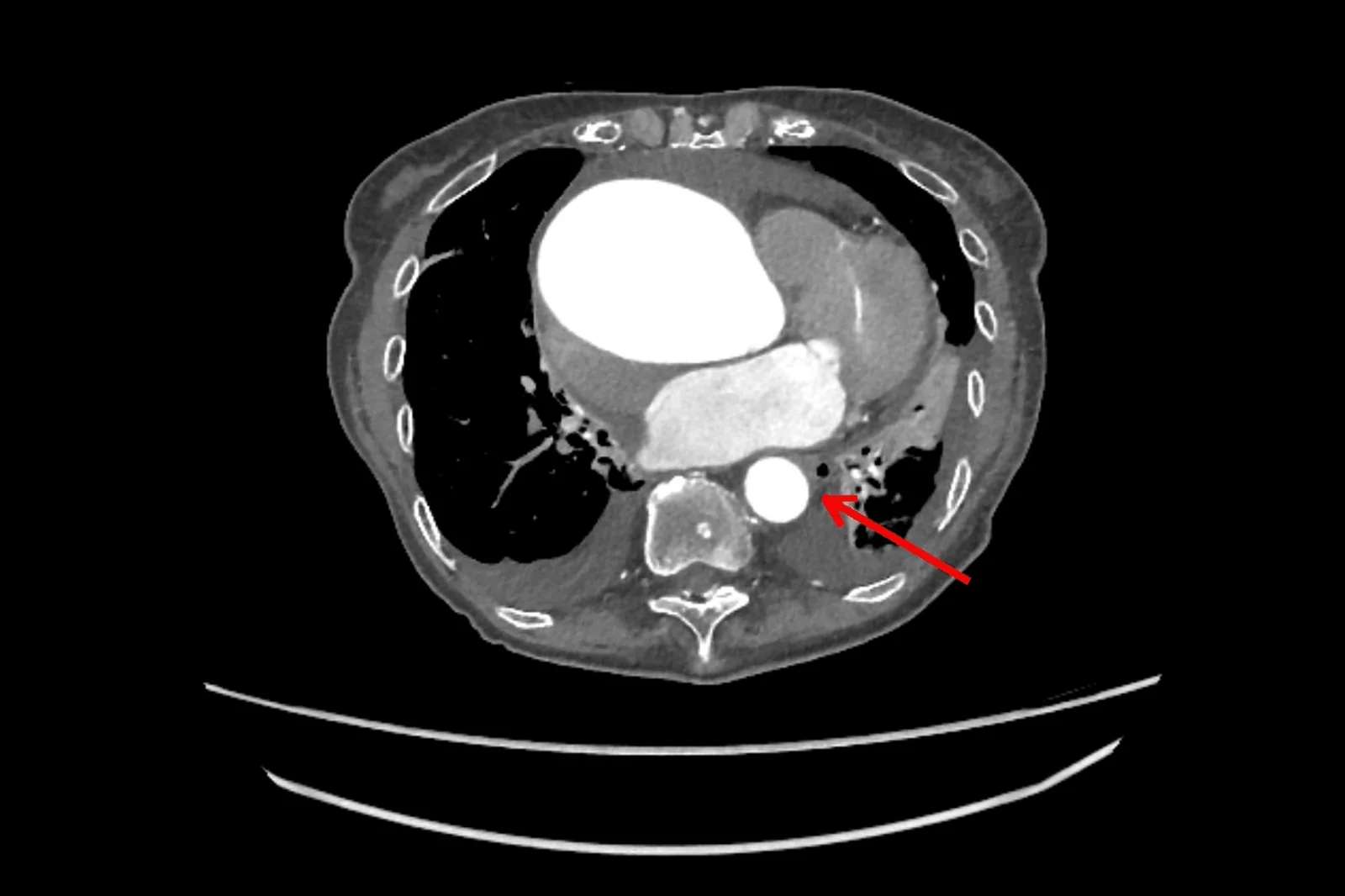
Axial CT angiogram at the level of the pulmonary trunk.

**Figure 10:**
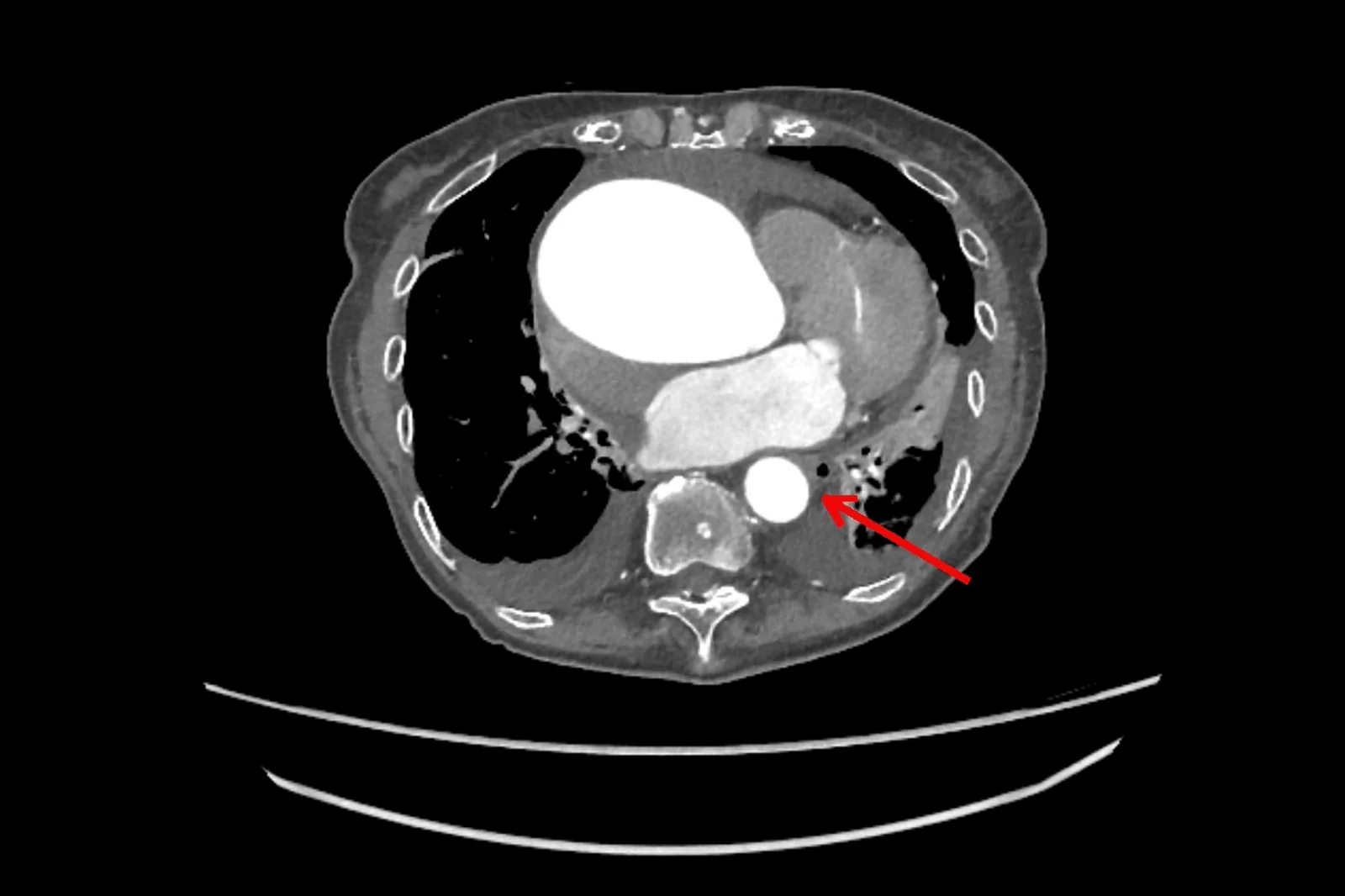
Axial CT angiogram at a more caudal level showing the aneurysm and pericardial effusion.

Coronal and sagittal reformatted images confirmed the extent of the aneurysm and its continuity with the normal-caliber aortic arch, descending thoracic aorta, and abdominal aorta (Figures 11-12).

**Figure 11:**
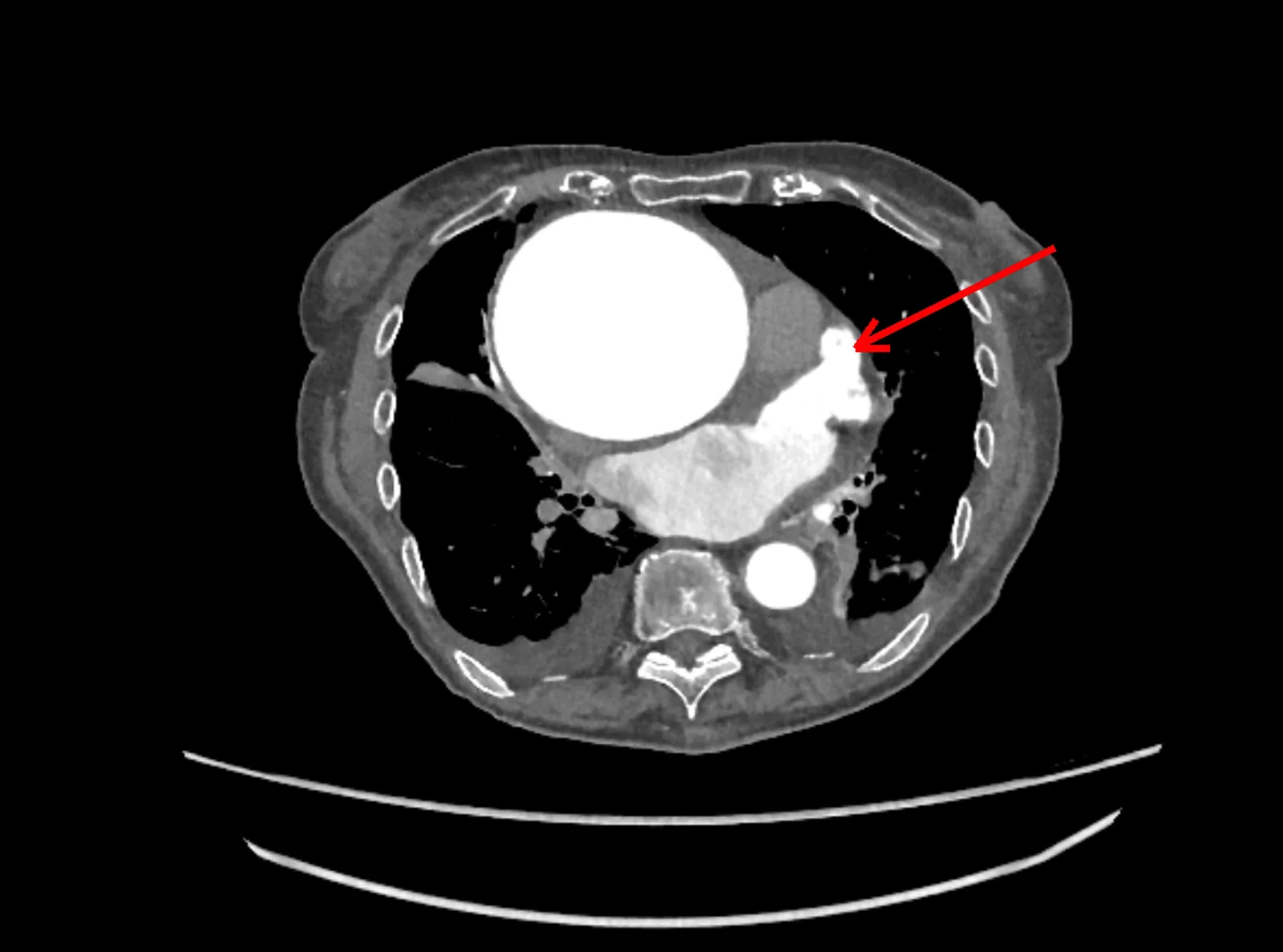
Coronal CT reformat showing the ascending aortic aneurysm and its relationship to the rest of the aorta.

**Figure 12:**
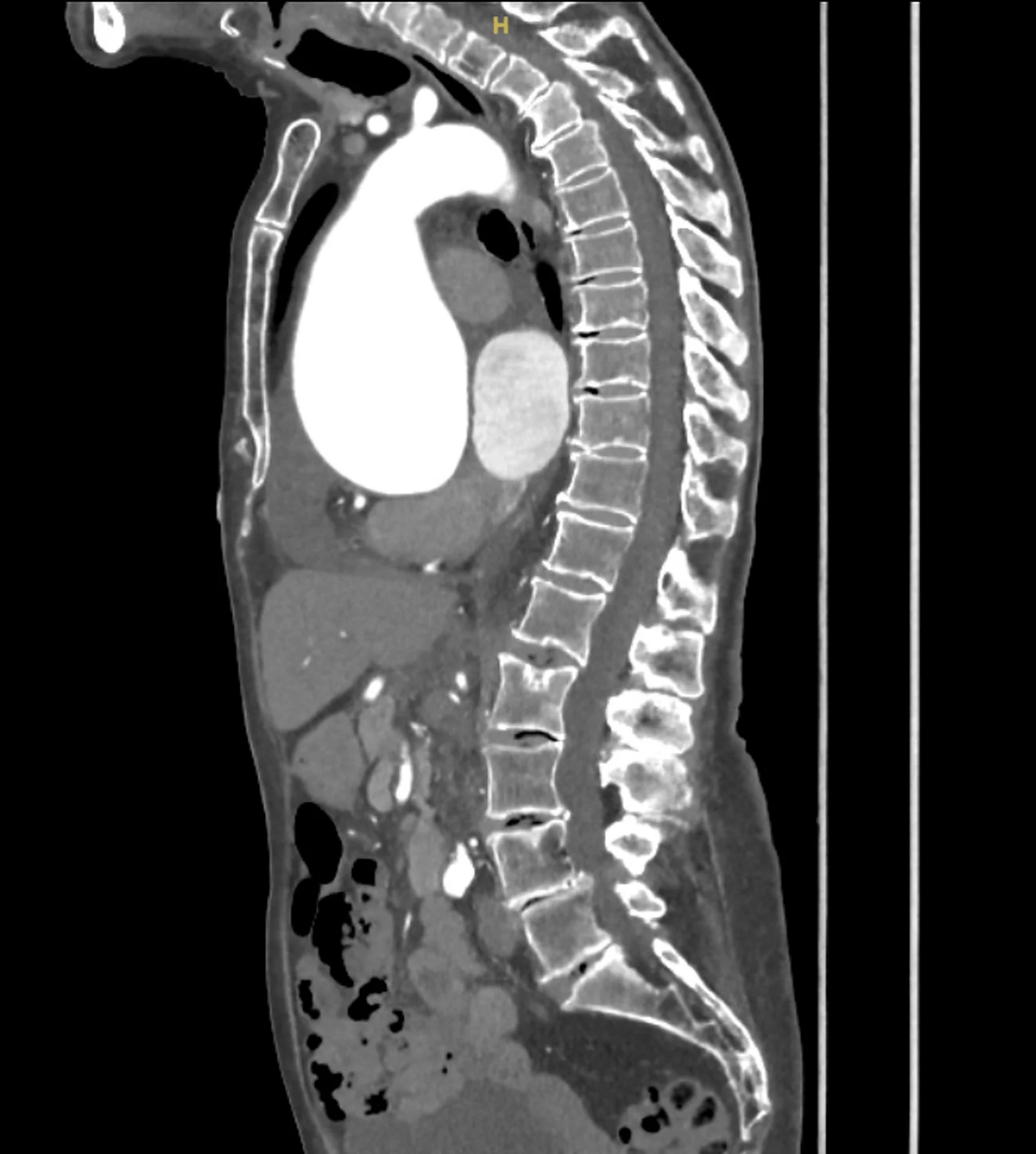
Sagittal CT reformat showing the anteroposterior extent of the aneurysm

Surgical assessment and shared decision-making

The patient was reviewed by the cardiothoracic surgical team. The decision not to proceed with open repair was based not on age alone, but on the combined effect of her advanced age, frailty, comorbidities, renal impairment, ventricular dysfunction, and the overall risk of a major operation. Following multidisciplinary discussion, nonoperative management was agreed upon.

The clinical team then held a detailed discussion with the patient about anticoagulation. The indication for apixaban 5 mg twice a day in the setting of atrial fibrillation was explained, as was the concern that anticoagulation could complicate a catastrophic bleeding event if the aneurysm ruptured. The patient had decision-making capacity and no evidence of cognitive impairment. After the potential benefits and risks had been discussed, she chose to continue apixaban. Her son was also informed of the decision.

Medical optimization and discharge

Lisinopril was initiated and gradually increased to 40 mg daily to improve blood pressure control and reduce hemodynamic stress on the aortic wall. Heart failure therapy, diuretics, and rate-control treatment were continued.

After five days of inpatient treatment, the patient was clinically stable enough for discharge. She received advice about maintaining good blood pressure control, avoiding heavy lifting and straining, and seeking urgent medical attention if she developed concerning symptoms. She was also advised that the apixaban dose should be reduced to 2.5 mg twice daily if her renal function deteriorated sufficiently to meet the relevant dose-reduction criteria.

Follow-up

At outpatient review two months after discharge, the patient reported that her dyspnea and lower-limb edema had improved. Her atrial fibrillation was rate-controlled on electrocardiography. Home blood pressure recordings showed systolic pressures ranging from 100 to 126 mmHg, with diastolic readings between 40 and 65 mmHg.

A repeat bedside echocardiogram showed no meaningful change in the aneurysm, which measured 9.4 cm. A small residual pericardial effusion remained. Left ventricular systolic function was still reduced, with an ejection fraction of approximately 40%, and significant aortic and mitral regurgitation persisted. Her treatment was continued, and she was referred to the community heart failure service for ongoing follow-up.

## Discussion

An aneurysm at the extreme of the reported size distribution

Ascending aortic aneurysms of this size are rarely encountered in contemporary practice. Regular surveillance and elective repair generally prevent aneurysms from reaching 9 cm or more [1]. In a widely cited analysis, Elefteriades described a “hinge point” at approximately 6 cm, beyond which the annual risk of rupture, dissection, or death increases substantially [2]. The aneurysm in this case was considerably larger than that threshold, yet it had not ruptured or dissected by the time it was identified.

The few comparable reports in the literature illustrate the range of possible clinical pathways. One report described an 85-year-old woman with an uncomplicated 9-cm ascending aortic aneurysm who underwent successful surgical replacement [3]. Another described a 73-year-old man with a 10-cm, heavily calcified ascending aortic aneurysm who presented with decompensated heart failure and atrial fibrillation and subsequently underwent surgery [4]. In contrast, reports describing a patient with an aneurysm of this size who was considered unsuitable for surgery from the outset, and whose immediate clinical problems were atrial fibrillation and heart failure, are limited.

The reason this patient reached a diameter of 9.3 cm without a previous diagnosis is uncertain. Long-standing hypertension was an important risk factor, and she had not previously undergone chest CT or echocardiography. Her presentation also demonstrates how a giant aneurysm may remain clinically silent until imaging is obtained for another reason. In this case, the symptoms that brought her to hospital were more consistent with arrhythmia and fluid overload than with an acute aortic complication.

Incidental detection through the heart failure workup

The aneurysm was identified because echocardiography was performed as part of the assessment of new-onset heart failure. The admission chest radiograph had already suggested an abnormal mediastinal contour, reported as an enlarged or mass-like mediastinal opacity. Similar radiographic findings have been described as an initial clue in other cases of giant ascending aortic aneurysm [4].

The discrepancy between the echocardiographic estimate of at least 7.5 cm and the CT measurement of 9.3 cm should not be interpreted simply as evidence that echocardiography is unreliable. In this case, the echocardiographer clearly documented that the aortic walls could not be adequately traced and appropriately recommended cross-sectional imaging. The more important lesson is that a numerical measurement should not be relied upon when the margins of the aorta are poorly visualized. When echocardiography suggests substantial ascending aortic dilatation but cannot define the aortic walls, CT or magnetic resonance imaging should be obtained before clinical decisions are based on the measurement [1].

Interacting pathophysiology

The patient’s heart failure was probably multifactorial. A rapid ventricular response from new-onset atrial fibrillation can precipitate heart failure, particularly in an older patient with reduced cardiovascular reserve. Loss of coordinated atrial contraction may further impair ventricular filling. In addition, the significant aortic regurgitation imposed a chronic volume load on the left ventricle.

The left ventricular ejection fraction of 40% should also be interpreted in the context of aortic regurgitation. In the presence of substantial regurgitant flow, the ejection fraction may appear less impaired than the underlying myocardial contractility because the left ventricle ejects blood both into the systemic circulation and backward through the incompetent valve. This is clinically relevant when assessing the severity of ventricular dysfunction and considering the likely contribution of the valve lesion to the patient’s symptoms.

The associated pericardial effusion required particular attention. Pericardial fluid is common in patients with heart failure, and the CT appearance was consistent with simple fluid rather than hemorrhage. Nevertheless, in a patient with a 9.3-cm ascending aortic aneurysm, any pericardial effusion raises concern for a contained leak or other aortic complication. The echogenic material seen within the effusion on echocardiography added to that concern. The effusion remained stable and did not cause hemodynamic compromise during the two-month follow-up period, making a heart failure-related effusion more likely, but the finding warranted close clinical and imaging surveillance.

Rate control and blood pressure targets in the context of aortic regurgitation

Rate control was achieved using digoxin and an increased dose of bisoprolol. Digoxin requires particular caution in older adults with chronic kidney disease because it is predominantly renally cleared and has a narrow therapeutic range. Dose selection and ongoing monitoring of renal function are therefore important in this setting [5].

Beta-blockers have traditionally been used cautiously in significant aortic regurgitation because a slower heart rate prolongs diastole and may theoretically increase the time available for regurgitant flow. However, observational data have not consistently supported the assumption that beta-blocker therapy is harmful. In a cohort of 756 patients with severe aortic regurgitation, beta-blocker treatment was associated with improved survival, although the study was observational and cannot establish a causal relationship [6]. The clinical question remains unsettled [7]. In this patient, beta-blockade had two clear purposes: controlling the ventricular response in atrial fibrillation and reducing hemodynamic stress on the aorta. The rationale for treatment should be documented explicitly in patients with competing physiological considerations.

Blood pressure control remains one of the principal components of medical management for thoracic aortic aneurysm because reducing arterial pressure can reduce wall stress [1]. Lisinopril was therefore increased to 40 mg daily. At follow-up, systolic pressures were well controlled, ranging from 100 to 126 mmHg. However, diastolic pressure was sometimes as low as 40 mmHg. Low diastolic pressure may accompany significant aortic regurgitation, but it may also reduce coronary perfusion, which is important in an older patient with biventricular systolic impairment. This case highlights the need to balance protection of the aortic wall against the risk of excessive diastolic blood pressure reduction. There is no single universally applicable blood pressure target for this clinical combination, so treatment must be individualized and reviewed over time.

The anticoagulation dilemma

The most difficult management decision in this case was whether to continue anticoagulation. The patient’s risk of thromboembolic stroke was high because of her age, hypertension, and heart failure, making the indication for anticoagulation in atrial fibrillation strong [8]. Against this benefit was the understandable concern that anticoagulation could worsen the outcome of an aneurysmal rupture.

An intact aortic aneurysm is not, by itself, an established contraindication to anticoagulation and is not listed as an exclusion to anticoagulation in contemporary atrial fibrillation guidance [8]. If rupture occurs, the prognosis is likely to be extremely poor regardless of anticoagulant status. In contrast, the risk of embolic stroke associated with untreated atrial fibrillation is clinically meaningful and potentially preventable. The decision in this case was therefore to continue apixaban after an individualized discussion rather than to withhold anticoagulation solely because of the aneurysm.

The process by which the decision was reached was as important as the treatment choice itself. The patient had capacity, received an explanation of the competing risks, and was allowed to express her preference. Her son was informed, and the discussion was documented. When surgery is not a realistic option, careful communication and shared decision-making become central components of active treatment rather than secondary considerations.

The patient was also given specific advice about possible apixaban dose reduction if renal function deteriorated. The dose-reduction criteria used in the ARISTOTLE trial require at least two of the following: age 80 years or older, body weight 60 kg or less, or serum creatinine of at least 133 µmol/L (1.5 mg/dL) [9,10]. She already met the age criterion but weighed 73 kg; a creatinine increase above the specified threshold would therefore provide her second criterion. Any conditional dosing plan should state these triggers clearly and ensure that the clinicians providing community follow-up are aware of them.

Frailty, age and the limits of intervention

The decision not to operate was not based on chronological age alone. It reflected the combined effect of age, frailty, renal disease, ventricular dysfunction, and other comorbidities. Frailty is independently associated with higher mortality, prolonged institutional care, and poorer outcomes after cardiac surgery [11]. A systematic review has likewise shown that preoperative frailty is associated with adverse outcomes extending beyond the immediate postoperative period [12]. Use of a formal instrument such as the Clinical Frailty Scale can provide a more transparent and reproducible basis for these discussions than age alone [13]. A formal frailty assessment, when available, can provide a more transparent and reproducible basis for discussing operative risk than chronological age alone. A formal frailty score was not documented; however, her baseline independence was broadly consistent with a Clinical Frailty Scale score of approximately 3.

Endovascular treatment of the ascending aorta remains highly specialized and is not a routine alternative to open repair. Dedicated devices are not widely available for this anatomical segment, and published experience remains limited, heterogeneous, and focused mainly on dissection, intramural hematoma, rupture, or treatment of postoperative complications rather than slowly enlarging aneurysms such as the one described here [14,15].

Nonoperative management should not be viewed as therapeutic abandonment. In this patient, it involved active blood pressure control, treatment of heart failure, careful rate control, anticoagulation, counseling about activity and warning symptoms, structured follow-up, and referral to community heart failure services. The goal was to provide the safest achievable care within the limits imposed by the patient’s overall condition and preferences. The clinical significance of this case lies in its demonstration that a giant ascending aortic aneurysm may remain clinically silent and be detected incidentally during evaluation of atrial fibrillation and heart failure. It also highlights the importance of confirming suspected aortic dilatation with cross-sectional imaging and using individualized, shared decision-making to balance anticoagulation, blood pressure control, and surgical risk when definitive repair is not feasible.

Strengths and limitations

A strength of this case is the detailed multimodality assessment, in which echocardiography raised suspicion of marked ascending aortic dilatation and CT angiography subsequently confirmed the aneurysm’s size, anatomical extent, and absence of dissection or rupture. The report also provides a clinically relevant account of management in a frail older patient who was not considered suitable for surgical repair, including the interaction between heart failure, aortic regurgitation, atrial fibrillation, blood pressure control, and anticoagulation. In addition, the case illustrates the importance of shared decision-making when the benefits and risks of treatment must be balanced against limited procedural options. Nevertheless, this report describes a single patient followed for only two months and therefore cannot establish the long-term outcome of nonoperative management of a giant ascending aortic aneurysm. Aortic measurements also varied according to the imaging modality: the initial echocardiographic estimate was at least 7.5 cm, whereas CT and subsequent bedside echocardiography measured between 9.2 and 9.4 cm. The CT measurement was considered the most reliable because the aortic margins were more clearly defined. No tissue sampling or genetic testing was performed, so the presumed contribution of long-standing hypertension remains a clinical inference rather than a confirmed etiology. These limitations should be considered when interpreting the clinical course and generalizing the findings to other patients.

## Conclusions

This case describes the incidental identification of a giant 9.3-cm ascending aortic aneurysm during the evaluation of new-onset atrial fibrillation and decompensated heart failure in an 88-year-old woman. Although the aneurysm was clinically silent and showed no evidence of dissection or rupture, its presence complicated the management of heart failure, aortic regurgitation, rate control, blood pressure, and anticoagulation. When echocardiography suggests substantial ascending aortic dilatation but provides inadequate visualization, CT or magnetic resonance imaging is essential for definitive assessment. In a frail older patient who is not suitable for surgical repair, management should remain active and individualized, incorporating blood pressure control, heart failure treatment, careful follow-up, and shared decision-making regarding anticoagulation. This case highlights that routine cardiac imaging performed for atrial fibrillation and heart failure may reveal life-threatening aortic disease and that, when surgery is not feasible, a clear discussion of risks, benefits, and patient preferences is central to appropriate care.
